# Aesthetic results in twin case undergoing transverse abdominoplasty versus Fleur-de-Lis abdominoplasty after massive weight loss

**DOI:** 10.1016/j.jpra.2024.07.016

**Published:** 2024-08-05

**Authors:** Shadi Javadian, Jais Oliver Berg

**Affiliations:** a The Department of Plastic and Reconstructive Surgery, Herlev University Hospital, in the Capital Centre of Massive Weight Loss Plastic Surgery, Copenhagen, Denmark; b Printzlau Private Hospital, Appointed Center of Massive Weight Loss Surgery in the Capital Region of Denmark, Hjortholmsvej 2, DK-2830 Virum, Denmark

**Keywords:** Abdominoplasty, Fleur-de-Lis abdominoplasty, Body contouring, Massive weight loss, Quality of life

## Abstract

This identical twin case demonstrates the aesthetic differences of two different body contouring procedures in alike patients. Body contouring improves physical, psychosocial, and sexual function therefore providing the best possible aesthetic outcome for massive weight loss patients is important. The Fleur-de-Lis pattern should be strongly considered when dealing with moderate to significant horizontal skin excess of the abdomen to obtain the best possible contouring and create an attractive female contour of hip and waist.

## Introduction

Patients presenting with a desire to undergo body contouring procedures after massive weight loss (MWL) continue to increase in number.^1^^,^^2^ Deformities of the abdominal contour following MWL are highly variable among patients, ranging from mild skin excess to multiple rolls.^3^ MWL patients often present with significant skin excess in both the horizontal and vertical vectors, and the vertical midline region may not be adequately treated with the traditional abdominoplasty technique.^1^ The solution to this can be the Fleur-de-Lis abdominoplasty (FdL), incorporating a vertical excisional component.^1^^,^^4^ This case emphasizes the importance of meticulous patient selection for each respective abdominoplasty technique and opens a discussion on the aesthetic results of the two techniques. What is the priority: less scarring or maximum body contouring?

## Case report

**Figure 1:**
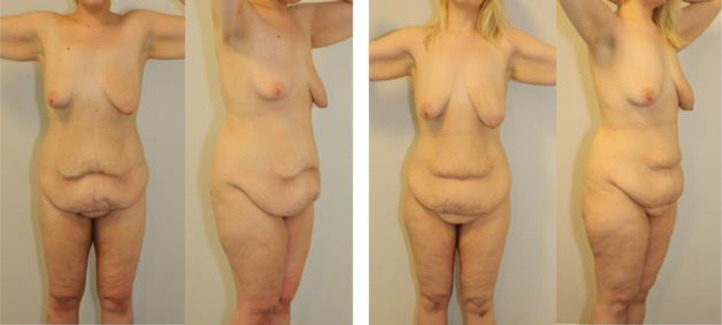
Preoperative status. Left: Twin A had lost 64 kg from 150 kg to 86 kg. BMI-loss from 47 to 27 kg/m^2^. EBWL 91%. Right: Twin B had lost 58 kg from 140 kg to 82 kg. BMI-loss from 45 to 26 kg/m^2^. EBWL 92%.

A pair of 23-year-old identical twins achieved stable MWL by lifestyle changes with a similar loss of excessive body weight (EBWL) of 91 % and 92 %, respectively. The abdominal contour changes were similar with vertical and horizontal (peri-umbilical) skin excess and a widened, ptotic mons (Figure 1); grade 2 according to the Danish Scale.^5^ The patients were healthy and non-smokers.

Twin A requested a low positioned horizontal scar and was offered a lower bodylift since a supra-gluteal ridge of excess tissue gave functional problems. She had 2.675 kg removed from the abdomen with the highest resection of 32 cm (12.6 inches) in the midline. Twin B had only problems with the abdominal tissue excess and was recommended a FdL. This was performed without undermining, with a maximal vertical resection width of 17 cm (6.7 inches), a maximum resection height of 19 cm (7.5 inches) in the midline, and 2.70 kg removed in total. No liposuctioning, rectus plasty or other plicatures of the musculoaponeurotic system were performed on either of the patients. The recoveries were uneventful, and subjectively both patients were happy and content with their respective result. Objectively no surgical corrections on the abdomen were needed at the 1-year follow-up.

## Results

**Figure 2:**
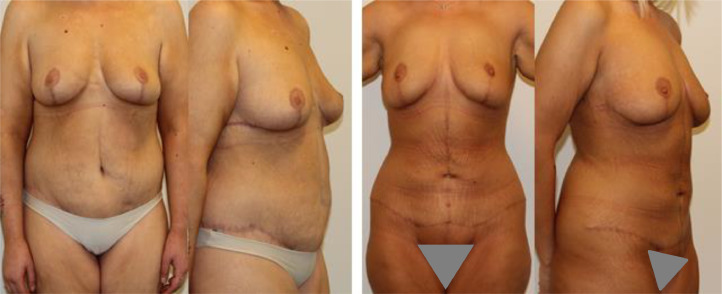
Postoperative status. Left: Twin A has a waist-hip ratio close to 1. Right: Twin B has a waist-hip ratio of 0.7.

Figure 2 shows the results one year postoperatively (three months after breast corrections). Twin A had some residual skin excess with two peri-umbilical columns of excess tissue and a vertical midline groove. Twin B had an aesthetically pleasing result with no significant skin excess. Unfortunately, for comparison reasons, there were differences in weight stability as twin A had gained 6 kg, and twin B was weight stable. If twin A had remained weight stable, she would probably have had less bulging of the subcutaneous fat, but the periumbilical horizontal skin excess with vertical columns would probably have appeared the same or looser.

## Discussion

Some plastic surgeons have reservations about the FdL technique due to an allegedly higher risk of complications compared to for example the traditional abdominoplasty procedure.^1^^,^^6^ In a retrospective and a prospective study, both evaluating the clinical outcomes and complications of the FdL technique and traditional abdominoplasty, including 56 and 499 patients respectively,^6^^,^^1^ no statistically significant difference in major complications was found. Only surgical site infections were significantly higher in the FdL cohort. Hence the authors conclude that FdL abdominoplasty can be performed as safely as the traditional abdominoplasty in MWL patients.

It is stated that the best candidates for FdL abdominoplasty are patients with epigastric skin laxity.^1^ This is a common perception.^4^^,^^6^^,^^7^ However, periumbilical horizontal skin excess may also be an indication^2^ as our case demonstrated. Different experiences, preferences, and surgical approaches are described and debated in the literature.^3^^,^^4^^,^^7^ Our case demonstrates the results of a low transverse scar abdominoplasty vs. a FdL abdominoplasty on *“the same patient”,* i.e., identical twins with the same body features and areas of skin excess, operated by the same plastic surgeon (JOB), with no other techniques used to improve the abdominal contours. It shows the powerful effects of the vertical resection in removing excess tissue in the horizontal vector of the abdomen and the superiority in creating an attractive female waist-to-hip ratio, when dealing with MWL patients who have both skin excess in the epigastric area and in the periumbilical area.

Another important aspect is the physical attractiveness achieved with body contouring. Although debated in the literature, the majority of authors agree that a waist-to-hip ratio of 0.70 is considered the most attractive in females and 0.75 in males.^8^ In our case, twin B had a waist-to-hip ratio of 0.7 whilst twin A had a ratio of 1. A cautious approach with the FdL design in male patients is advisable since the procedure has the potential of a non-desirable waist-to-hip ratio if the skin is tightened at its maximum in the horizontal direction.

In a systematic review and meta-analysis, Toma et al. (2018) found statistically significant improvements in physical, social and psychological functioning in MWL patients undergoing body contouring surgery.^9^ Moreover, the patient-reported scar evaluation is found similar in the FdL abdominoplasty group and traditional abdominoplasty group.^6^ This also applies to our case as both patients were satisfied with the postoperative aesthetic result and neither had complaints about the scars.

Considering the abovementioned factors of improvement in patient-reported outcomes, providing the best possible aesthetic outcome for MWL patients becomes crucial. The FdL incision is known to create a more enhanced correction of the contour deformities in some bariatric patients, as it addresses vertical and horizontal skin and soft tissue excess as opposed to traditional abdominoplasty which tends to neglect midline/ supraumbilical tissue excess.^4^^,^^6^ This was also shown in our twin-case when comparing the postoperative results. Our patients were both satisfied and content with the surgical results; however, objectively, one could argue that the body contouring of twin B was more aesthetically pleasing due to obtaining an ideal waist-to-hip ratio. Hence, our case demonstrates the importance of meticulous selection of patients when deciding what type of body contour procedure should be performed. However, the empirical results reported in this paper should be viewed in the light of sample size limitations. This could have led to an overestimation of the results and future research should reconfirm these findings by conducting larger-scale case studies.

## Conclusion

In conclusion, this case of identical twins with similar excess skin changes after MWL compares the two main abdominoplasty designs. It highlights the visual effects of the vertical resection in the FdL procedure and the superiority in creating an attractive female contour of the hip and waist. The FdL pattern in abdominal body contouring is a safe and effective technique for properly selected massive weight-loss patients. The more scarring from the FdL procedure is, in our experience, typically well tolerated by patients if they are well-informed preoperatively, since they tend to focus on the resultant contours.

We advocate that the FdL pattern should be considered for female MWL patients with moderate or significant horizontal (peri-umbilical) skin excess to achieve the best possible contouring results in a single procedure.

## Funding

This research did not receive any specific grant from funding agencies in the public, commercial, or not-for-profit sectors.

## Ethical approval

Not required.

## Declaration of competing interest

The authors report no conflicts of interest. The authors alone are responsible for the content and writing of the paper.
